# LXR agonist modifies neuronal lipid homeostasis and decreases PGD2 in the dorsal root ganglia in western diet-fed mice

**DOI:** 10.1038/s41598-022-14604-0

**Published:** 2022-06-24

**Authors:** Nadia Elshareif, Chaitanya K. Gavini, Virginie Mansuy-Aubert

**Affiliations:** Cell and Molecular Physiology, Stritch School of Medicine, Loyola University Chicago, Maywood, IL 60153 USA

**Keywords:** Cellular neuroscience, Somatic system

## Abstract

The prevalence of peripheral neuropathy is high in diabetic and overweight populations. Chronic neuropathic pain, a symptom of peripheral neuropathy, is a major disabling symptom that leads to a poor quality of life. Glucose management for diabetic and prediabetic individuals often fail to reduce or improve pain symptoms, therefore, exploring other mechanisms is necessary to identify effective treatments. A large body of evidence suggest that lipid signaling may be a viable target for management of peripheral neuropathy in obese individuals. The nuclear transcription factors, Liver X Receptors (LXR), are known regulators of lipid homeostasis, phospholipid remodeling, and inflammation. Notably, the activation of LXR using the synthetic agonist GW3965, delayed western diet (WD)-induced allodynia in rodents. To further understand the neurobiology underlying the effect of LXR, we used translating ribosome affinity purification and evaluated translatomic changes in the sensory neurons of WD-fed mice treated with the LXR agonist GW3965. We also observed that GW3965 decreased prostaglandin levels and decreased free fatty acid content, while increasing lysophosphatidylcholine, phosphatidylcholine, and cholesterol ester species in the sensory neurons of the dorsal root ganglia (DRG). These data suggest novel downstream interplaying mechanisms that modifies DRG neuronal lipid following GW3965 treatment.

## Introduction

Peripheral Neuropathy (PN) is a common complication in prediabetic and obese individuals that causes hypersensitivity, chronic pain and perturbances in sensory perception^1,2^. Effective interventions are available to address obesity and associated dysregulated metabolic pathways, however, limited options are available to effectively treat neuropathic pain^3,4^. We and others have shown that upon western-diet (WD) nutrition containing high fat, high sucrose and high cholesterol, mice develop features of neuropathic pain including nerve fiber degeneration, an increase in thermal and mechanical sensitivity, and neuronal hyperexcitability^5–7^. Loss of nerve fiber density and pain phenotypes have been specifically linked to the hyperexcitability of Na_v_1.8-positive dorsal root ganglia (DRG) neurons, which make up approximately 75% of the sensory neuron population^7,8^. A growing body of evidence points towards lipid dysregulation in the peripheral nervous system (PNS) as one of the culprits for the pathogenesis of PN, however, this relationship has yet to be fully investigated in sensory neurons^9,10^. In the past, transcriptomic analysis of peripheral nerves (sural and sciatic nerves) have revealed that lipid pathways (including LXR pathways) may contribute to pain and neuropathy in many models including diet-induced obesity models or genetic models of obesity^11^. Lipidomics on the sciatic nerve of db/db mice has also determined changes in lipid composition, namely increases in nerve free fatty acids and triglycerides^12^.

Liver X Receptors (LXRs) are nuclear transcription factors that regulate lipid homeostasis, inflammation, and phospholipid remodeling^13^. We have previously published that neuronal LXR activation with the synthetic agonist GW3965 protects sensory neurons from obesity-induced Endoplasmic Reticulum (ER) stress, resulting in decrease of pain indices commonly observed in mice fed a WD^5^. Treatment of mice lacking LXR in Na_v_1.8-positive neurons did not alleviate the pain phenotype observed in WD-fed mice, demonstrating that the agonist acts through LXR in sensory neurons to improve pain^5^. Despite these findings, the molecular mechanism linking neuronal LXR activation and the alleviation of pain in obese rodent models is still unclear.

LXR activation changed the expression of Lysophosphatidylcholine Acyltransferase 3 (LPCAT3) in sensory neurons^14^. In liver cells, LPCAT3 modulated the insertion of arachidonic acid into membrane phospholipids, ER stress, and inflammation in the context of obesity^15,16^. Interestingly, arachidonic acid is a known precursor of the prostaglandin synthesis pathway. Prostaglandins are lipid-derived mediators of neuroinflammation^17–19^. Prostaglandin D2 (PGD2) is also produced by neuronal cells and is involved in sensory pain mechanisms due to its pro-nociceptive properties^18,20,21^. PGD2 has been specifically identified in Na_v_1.8-expressing sensory neurons to modulate pain pathways, along with its other isoform Prostaglandin E2 (PGE2)^20^. LXR activation has been shown to directly represses Cyclooxygenase-2 (COX-2) gene expression and downstream prostaglandin synthesis in cartilage explants of a rat osteoarthritis model^22^. Similar observations were made in inflammatory macrophages^23^. Using translating ribosome affinity purification (TRAP) to specifically isolate mRNA in translation of Na_v_1.8- expressing sensory neurons, we observed that GW3965 treatment regulates various pathways and transcripts involved in maintaining neuronal function and most of all cellular lipid homeostasis. Interestingly, GW3965 decreased prostaglandin levels and modified the lipid composition of primary DRG sensory neurons of obese mice. We observed a decrease in free fatty acid content and an increase in lysophosphatidylcholine, phosphatidylcholine, and cholesterol ester species. Our data provides evidence for putative lipid signaling that may underlie the improvement in pain upon LXR activation.

## Results

### Western diet feeding induces translatomic changes in DRG sensory neurons

**Figure 1:**
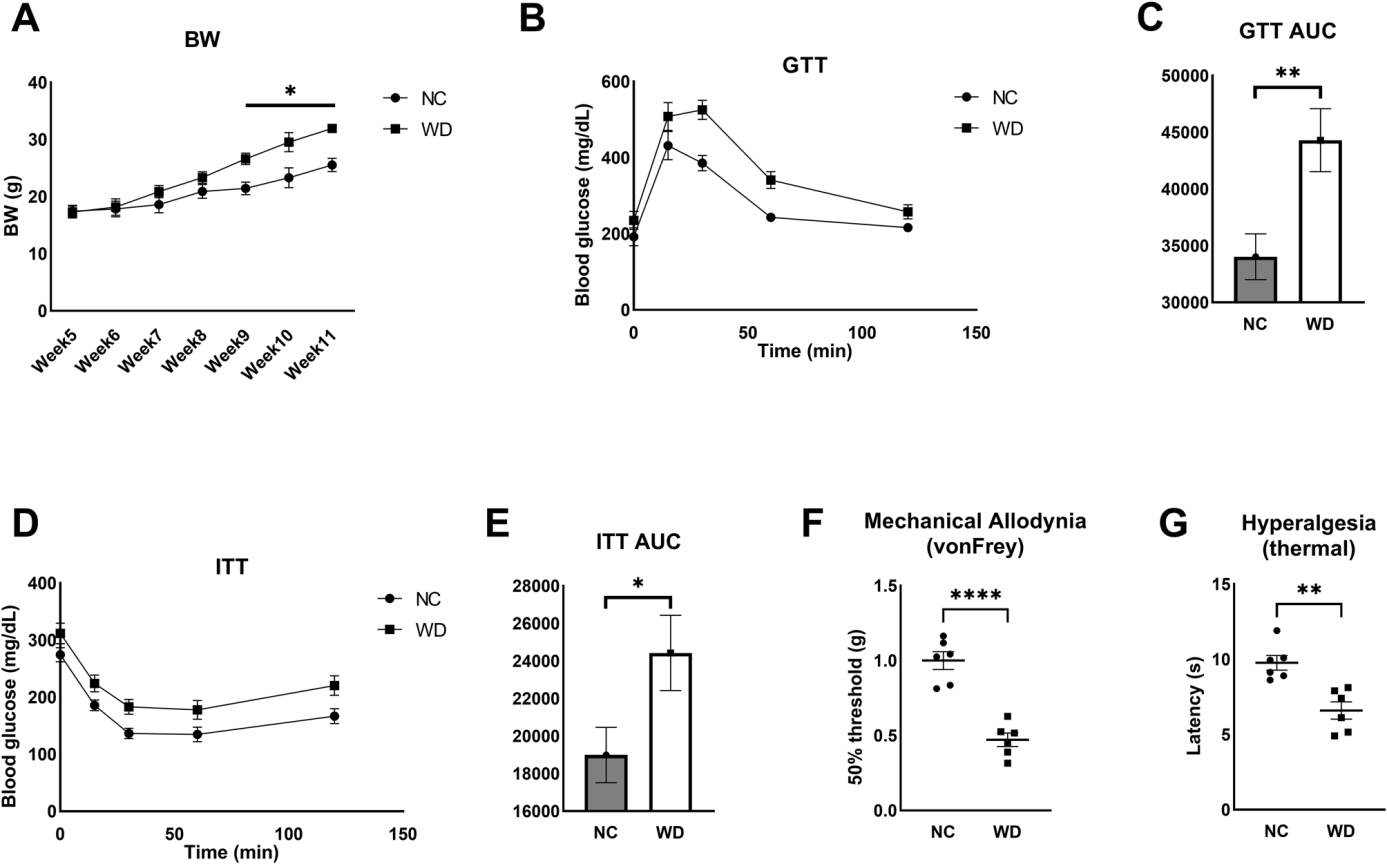
WD-fed mice develop glucose intolerance, insulin resistance and neuropathic pain. (**A**) Body weights (BW) taken over a period of 11 weeks of NC and WD-fed mice. (**B**) Glucose tolerance test and area under the curve (**C**). (**D**) Insulin tolerance test and area under the curve (**E**). (**F**) Von Frey behavioral test for mechanical allodynia. (**G**) Thermal behavioral test for hyperalgesia. Two-sample t-test, *p < 0.05, **p < 0.005, ****p < 0.0001.

**Figure 2:**
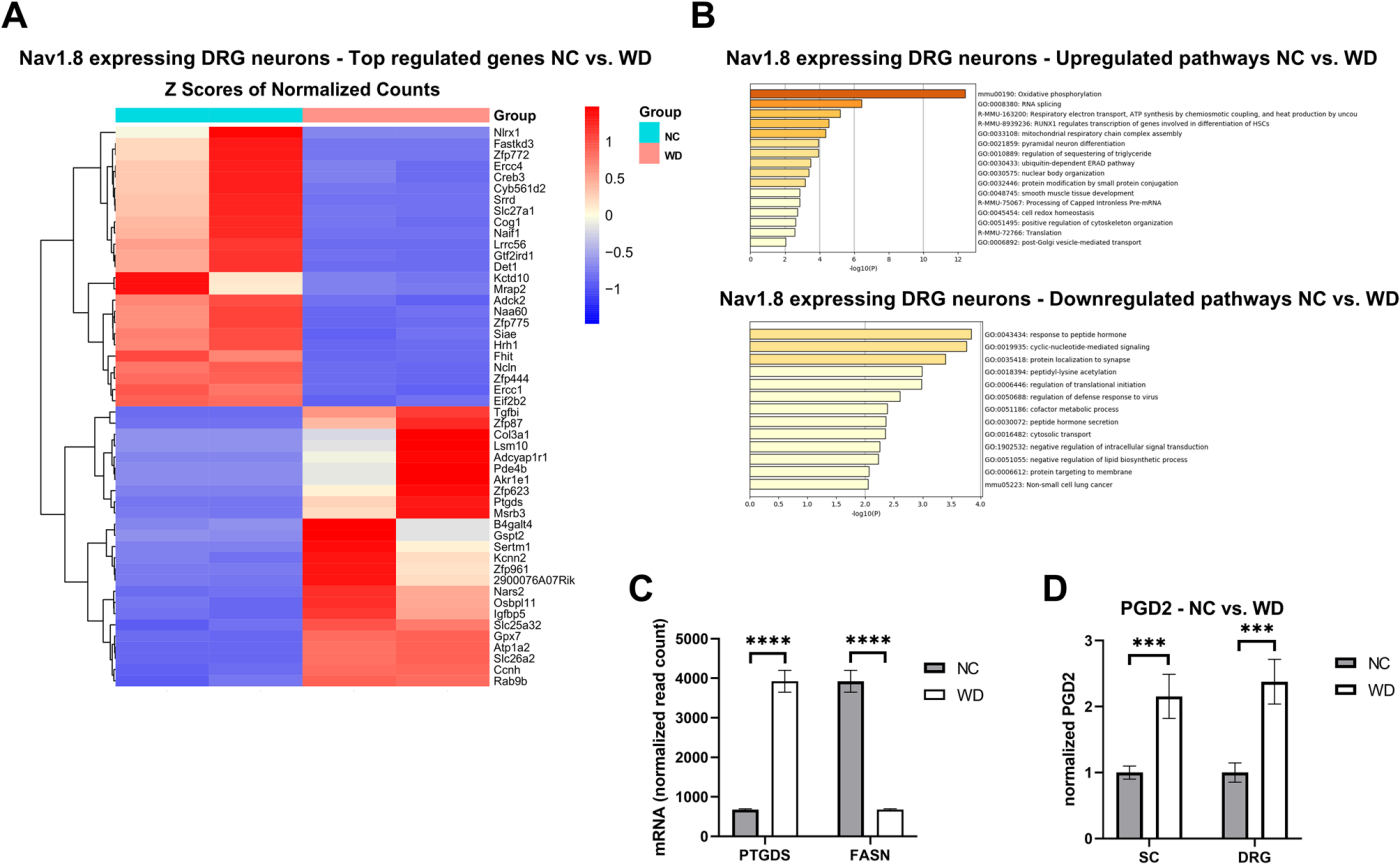
WD-fed mice display translatomic changes in DRG sensory neurons. (**A**) Heat map of top 50 regulated transcripts of Na_v_1.8-expressing sensory neurons of NC or WD-fed mice (n = 2 biological replicates/group, 3 DRG pooled from 3 mice, 9 DRG/replicates). (**B**) Pathway analysis of upregulated and downregulated transcripts. (**C**) mRNA read counts. FDR-adjusted p- values, ****p < 0.0001. Values are mean ± SEM. (**D**) normalized levels of PGD2 in the SC and whole DRG (n = 6 DRGs/ biological replicate) from mice fed either NC (n = 6) or WD (n = 7) for 11 weeks. Values are mean ± SEM. Two-sample t-test, ***p < 0.0005.

After 11 weeks of WD, mice developed features of metabolic syndrome concomitant with features of somatosensory dysfunction (Fig. 1 and as published previously)^5,24^. Mice on WD gain significantly more weight at 9 weeks of age compared to mice on the normal chow diet (Fig. 1A). They develop other characteristics of obesity including glucose intolerance and insulin resistance (Fig. 1B-E). This WD feeding paradigm induces pain phenotypes concurrent with neuropathic pain, including mechanical allodynia and hyperalgesia (Fig. 1 F, G). In order to identify translatomic changes induced by WD in sensory neurons of the DRG, we used RiboTag+/+:Na_v_1.8Cre+/- mice, which encodes an HA-tagged ribosomal protein (Rpl22) used to isolate the mRNA in translation specifically from Na_v_1.8-expressing sensory neurons as done previously^5^. We performed RNA sequencing on purified mRNA in translation from sensory neurons of mice fed WD or NC for 11 weeks^5^. We identified 344 transcripts differentially expressed in sensory neurons of WD- compared with NC-fed mice (Supplemental excel file 1), with the 50 most significantly dysregulated transcripts highlighted in Fig. 2A. Consistent with previous report^5^, WD feeding upregulated transcripts including Glutathione Peroxidase 7 (GPX7) and Methionine Sulfoxide Reductase B3 (MSRB3) that are involved in mitigating persistent ER stress and associated Unfolded Protein Response (Fig. 2A)^25,26^. We also identified upregulated transcripts involved in triglyceride sequestering, including Oxysterol Binding Protein Like 11 (OSBPl11), regulating triglyceride storage and sterol transport (Fig. 2A)^27,28^. Metascape analysis revealed pathway enrichment corresponding WD-induced ER stress, including oxidative phosphorylation, respiratory electron transport, and mitochondrial respiratory chain complex assembly (Fig. 2B). Of downregulated pathways and transcripts, we observed a downregulation in peptide hormone response, including genes involved in neuronal metabolism, including Melanocortin 2 Receptor Accessory Protein 2 (MRAP2) and fatty acid transport protein (SLC27A1), an insulin-dependent fatty acid transport protein (Fig. 2B)^29–31^. We also observed the downregulation of lipid processing and very-low density lipoprotein synthesis pathways, including fatty acid synthase (FASN) transcript that was downregulated by 4-fold (Fig. 2C, supplemental excel file 1). Interestingly, we found that prostaglandin D2 synthase (PTGDS), responsible for PGD2 synthesis, was one of the top five upregulated transcripts with a 5-fold increase in the DRG sensory neurons of WD-fed mice compared with NC (Fig. 2C). Given that PTGDS was upregulated in DRG sensory neurons of mice WD-fed, we then sought to determine the concentration of PGD2 in the spinal cord (SC) and DRG of WD and NC-fed mice, where prostaglandins and prostaglandin mediators of synthesis such as COX-2 are predominantly expressed in murine models of inflammation^18^. As shown in Fig 2D, compared to NC mice, there was a significant increase of PGD2 in SC and DRG of WD-fed mice (Fig. 2D).

### GW3965 treatment leads to translatomic changes in sensory neurons

**Figure 3:**
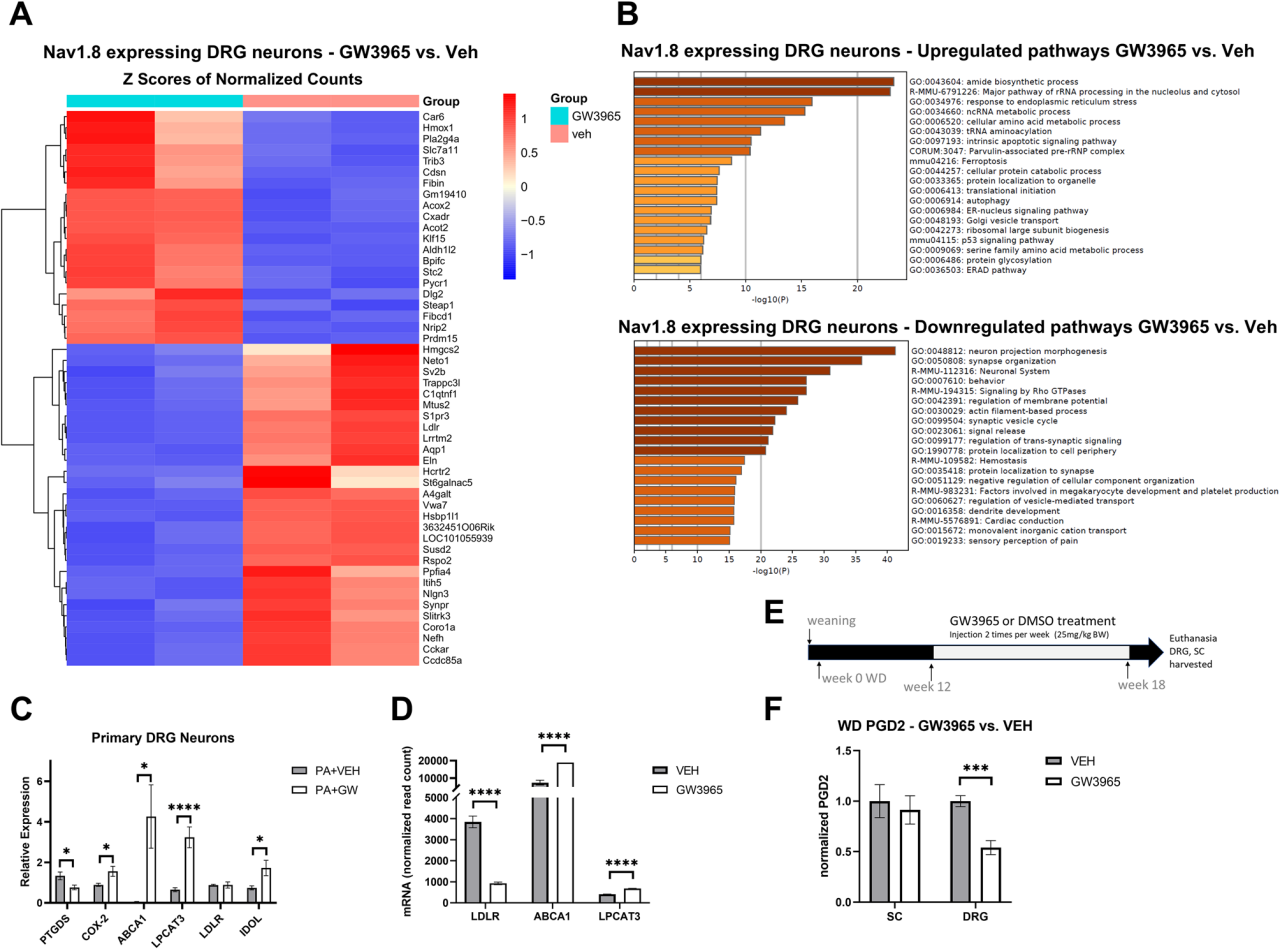
LXR agonist GW3965 induces translatomic changes in sensory neurons. (**A**) Heat map of top 50 dysregulated transcripts of Na_v_1.8-expressing sensory neurons from mice treated with either GW3965 (25 mg/kg BW) or vehicle (n = 2 biological replicates/group, 3 independent cultures pooled per group). (**B**) Pathway analysis of upregulated and downregulated transcripts. (**C**) mRNA levels of LXR target genes in primary DRG neurons treated with 250* μM* palmitate followed by vehicle or 15* μM* GW3965. (**D**) mRNA read counts of LXR target genes. (**E**) Eight week-old mice were placed on Western diet (WD) for 18 weeks. At week 12 of WD, mice were subjected to intraperitoneal injections of either GW3965 or DMSO twice a week for seven weeks. Mice were then euthanized, DRG and SC were harvested from each animal. (**F**) Normalized levels of PGD2 in the SC and whole DRG (n = 6 DRGs/biological replicate) from WD-fed mice treated GW3965 (25 mg/kg BW) or vehicle. Values are mean ± SEM. Two-sample t-test, *p < 0.05, ***p < 0.0005.

Because LXR activation modulates sensory neuron function and prostaglandin levels, we sought to identify all transcripts changed in the sensory neurons by GW3965. To this end, we isolated neurons from DRG of RiboTag+/+:Na_v_1.8Cre+/- mice and treated with 15*μM* of GW3965. We then purified mRNA in translation from Na_v_1.8-expressing neurons and performed RNA sequencing (Supplemental excel file 2). Figure 3A represents the top 50 significantly changed transcripts along with associated pathways revealed by Metascape analysis in Fig. 3B. Data revealed that GW3965 led to changes in transcripts involved in amide biosynthesis and amino acid metabolic pathways (Fig. 3B). GW3965 stimulation also increased transcripts involved in the response to lipid-induced ER stress, including Acyl-CoA Oxidase 2 (ACOX2), Acyl-CoA thioesterase 2 (ACOT2) (Fig. 3A,B)^32^. The transcript 3-hydroxy-3-methylglutaryl coenzyme A synthase (HMGCS2), however, decreased with GW3965 treatment (Fig. 3B). Aldehyde Dehydrogenase 1/2 (ALDH1/2) and stanniocalcin 2 (STC2) were also notably increased and are involved in fatty acid oxidation and oxidative stress (Fig. 3A,B). We also observed changes in mRNA in translation of cultured primary DRG neurons under saturated fatty acid conditions for LXR target genes that are crucial in regulating neuronal lipid homeostasis and transport, including LPCAT3, ATP Binding Cassette Subfamily A Member 1 (ABCA1), and IDOL, inducible degrader of the low-density lipoprotein receptor (LDLR) in independent experiments (Fig. 3C). LXRs inhibits the LDLR pathway through transcriptional induction of IDOL, an E3 ubiquitin ligase that triggers ubiquitination of the LDLR on its cytoplasmic domain, thereby targeting it for degradation^33^. Under these conditions, GW3965 decreased PTGDS mRNA levels by almost half (Fig. 3C). The LXR canonical genes ABCA1 and LPCAT3 were significantly increased after GW3965 treatment (Fig. 3D). LXR is a negative regulator of LDLR, and we also observed this modulation in sensory neurons where GW3965 downregulated its transcript by 2-fold (Fig. 3D).

Given our data, we hypothesized that LXR activation could modulate the level of PGD2 in the PNS of WD-fed mice. We injected mice with the LXR agonist GW3965 (25mg/kg BW) or vehicle, two times a week for seven weeks after 12 weeks on the diet paradigm (Fig. 3E). As previously reported, this paradigm did not lead to increase in triglyceride production by the liver, however, i.p. injection of GW3965 prevents the development mechanical allodynia^5^. This observation was LXR-specific, as we have previously published that Na_v_1.8-specific knockout of LXRs exacerbates both mechanical sensitivity and thermal nociception in WD-fed mice^5^. As shown in Fig. 3F, GW3965 treatment decreased the level of PGD2 by half in the DRG of WD-fed mice compared to vehicle treatment (Fig. 3F). Despite an increase in PGD2 in the SC of WD-fed mice, where prostaglandins are predominantly produced, there was no significant change in SC PGD2 of WD-fed mice injected with GW3965 (Fig. 3F).

### GW3965 modulates neuronal lipid composition

LXRs are well characterized in literature to modulate lipid homeostasis and phospholipid remodeling through the direct regulation of its target genes. Given the dysregulated pathways modulated by GW3935, we tested whether GW3965 modifies neuronal lipid homeostasis in DRG primary neurons. To this end, we comprehensively characterized the lipid species of primary DRG neurons treated with 15 *μM* GW3965 or vehicle using lipidomic experiments.

**Figure 4:**
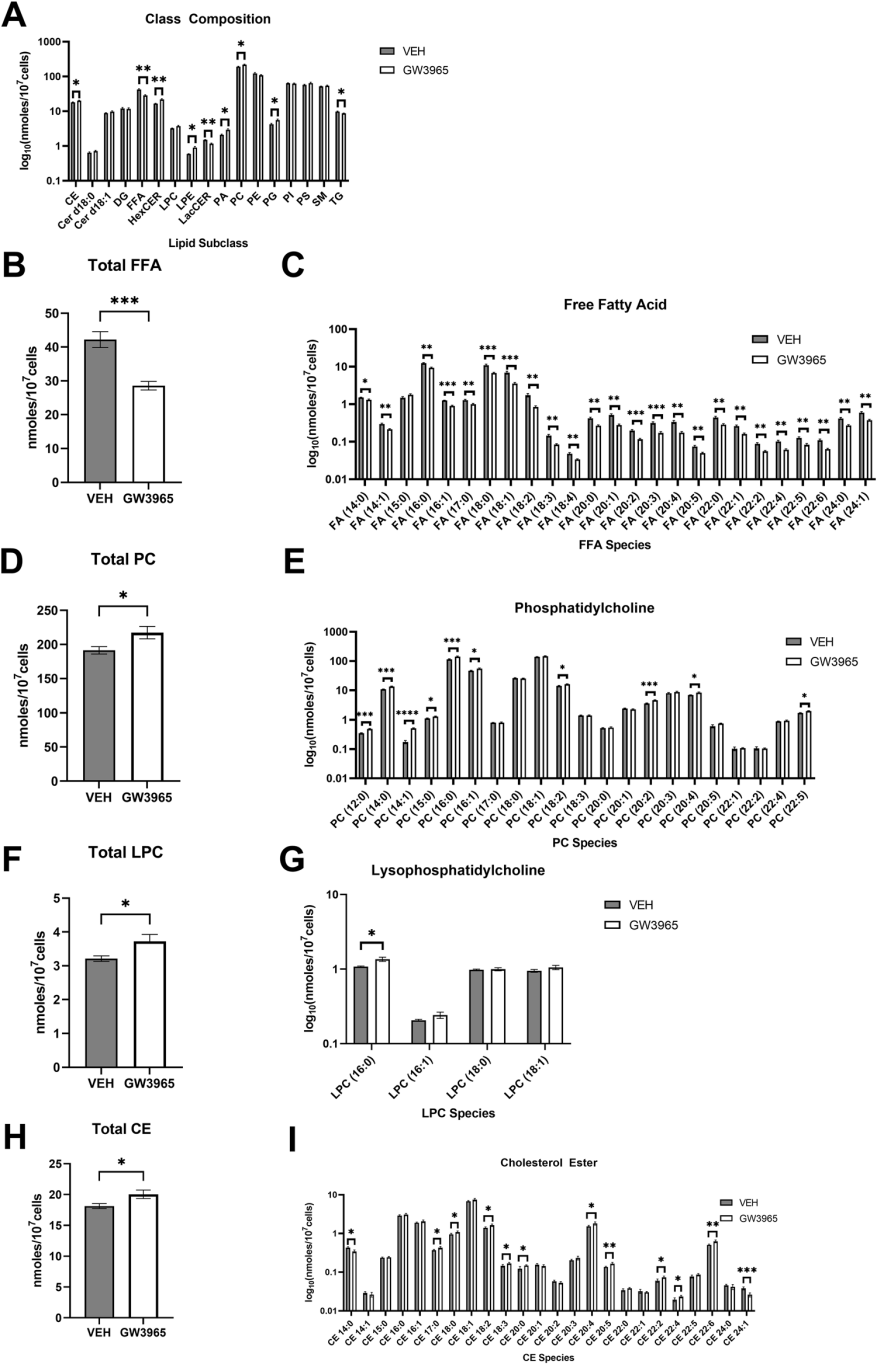
LXR agonist GW3965 modulates neuronal lipid composition. The concentration of various lipid subclasses (CE, cholesterol ester; Cer d18:1, ceramide; Cer d18:0, dihydroceramide; DG, diacylglycerol; FFA, free fatty acid; HexCER, hexocyl ceramide; LPC, lysophosphatidylcholine; LPE, lysophosphatidylethanolamine; LacCER, lactosyl ceramide; PA, phosphatidic acid; PC, phosphatidylcholine; PE, phosphatidylethanolamine; PG, phosphatidylglycerol; PI, phosphatidylinositol; PS, phosphatidylserine; SM, sphingomyelin; TG, triacylglycerol) (**A**) in primary Na_v_1.8- expressing DRG neurons treated with vehicle or 15* μM* GW3965 including (**B**) total FFA and FFA species (**C**), (**D**) total PC and PC species (**E**), (**F**) total LPC and LPC species (**G**), and (**H**) total CE and CE species (**H**) (n = 5 biological replicates/group). Values are mean ± SEM. Two sample t-test, *p < 0.05, **p < 0.005, ***p < 0.005.

GW3965 induced a variety of changes in lipid abundance across various lipid subclasses in primary DRG neurons (Fig. 4A). GW3965 significantly decreased almost all free fatty acids in the neurons of the DRG, suggesting their incorporation into other lipid species for long term storage and maintenance of lipid homeostasis (Fig. 4B). The most abundant fatty acids foundin the DRG were palmitic acid (16:0), stearic acid (18:0), and oleic acid (18:1), which are all fatty acids obtained from dietary fats and endogenous synthesis (Fig. 4C). Palmitic acid and oleic acid decreased in the sensory neurons upon treatment with GW3965 (Fig. 4C). There were no significant changes in the concentration of pentadecylic acid (15:0), an odd-chained fatty acid minimally obtained from diet (Fig. 4C)^34^. Of all lipid subclasses, phosphatidylcholine (PC) was the most abundant in primary DRG neurons and increased in abundance upon LXR activation (Fig. 4A,D). Notably, we observed a significant increase in arachidonoyl (20:4)- and linoleoyl (18:2)-PC species (Fig. 4D). Of all PC species, lauryl (12:0)-, myristyl (14:0)-,myristoyl (14:1)-, palmityl (16:0)-PC, which are all saturated fatty acids commonly incorporated into phospholipids, had the most significant increase in concentration upon treatment with GW3965 (Fig. 4E). Lysophosphatidylcholines are lipid substrates of Lpcat3 and serve as mediators of lipid metabolism^35,36^. Interestingly, we observed a significant accumulation of overall LPC species in GW3965-treated primary DRG neurons, particularly palmitic acid (LPC 16:0), a substrate in Lpcat3 for lipid remodeling (Fig. 4F,G)^35^.

Free cholesterol is converted into cholesterol esters with roles in cell maintenance and lipid trafficking. In membranes, cholesterol esters are largely responsible for the aggregation of lipid droplets and membrane raft dynamics. LXR activation increases various cholesterol ester concentrations in the DRG (Fig. 4H). We notably observed a significant increase in cholesterol esters (CE) conjugated with linoleic (CE 18:2) and arachidonic acid (CE 20:4), which are known substrates of LXR-induced lipid remodeling by the target gene LPCAT3 that were some of the most abundant (Fig. 4I). LXR may be modulating prostaglandin synthesis indirectly through the modulation of neuronal lipid homeostasis.

## Discussion

Obesity and related complications are the cause of almost half of cases of PN^37,38^. Lipid dysfunction as a result contributes to the pathogenesis of PN neuropathic pain^10,39^. We and others have demonstrated that WD-induced ER stress and mitochondrial dysfunction in the DRG^5,40,41^. Our current data provides a picture of the translatomic changes induced by WD on murine sensory neurons. We confirmed ER stress and mitochondrial dysfunction, accompanied by the upregulation of triglyceride sequestering pathways, which has been identified as a mechanism to prevent neuronal lipotoxicity^27,42^. We also identified a decrease in FASN transcript which in other tissue types, resulted in compromised mitochondrial energetics that may potentially exacerbate mitochondrial dysfunction^43^. Evidence of these dysregulated transcripts support the hypothesis that lipid signaling in the sensory neurons is a key contributor to nerve dysfunction. Our previous data demonstrated that LXR activation in sensory neurons protects against cellular stress induced by WD^5^. Here, we identified various pathways underlying LXR activation and its protective effect, including oxidative phosphorylation, lipid homeostasis, and the sensory perception of pain. While the causes of pain likely depends on multiple perturbed pathways, inflammation may remain a central contributor in obesity and upon WD nutrition^1,44,45^. PGD2 is of interest as it is abundant in neuronal cells and involved in sensory pain mechanisms^18,20,21^. Both neuropathic pain and inflammation are associated with metabolic disease states^44^. Prostaglandin D2 synthesis has been previously characterized in sensory neurons, and is involved in peripheral sensitization and neuropathic pain upon autocrine activation^20,21,46^. Here, we identified the upregulation of Ptgds transcripts accompanied by PGD2 levels in sensory neurons, while other isoform-specific enzymes were not detected. We demonstrated that LXR activation decreased PGD2 levels in the DRG, suggesting that LXR may be modulating prostaglandin synthesis indirectly through the modulation of neuronal lipid homeostasis under high fat conditions. LXR activation may reduce PGD2 levels in the DRG of WD-fed mice potentially via the lipid remodeling capabilities of Lpcat3. Indeed, LPCAT3 catalyzes the incorporation of polyunsaturated fatty acids such as arachidonic acid (20:4) and linoleic acid (18:2), into membrane phosphatidylcholine species at the sn-2 position^14,15^. Interestingly, linoleic acid and arachidonic acid may accumulate in sensory neurons, due to an increased in elevated phospholipase A2 (Pla2) activity in obesity-induced models^47^. The decrease in arachidonic and linoleic acid-containing phosphatidylcholine species upon LXR treatment may be occurring because of LPCAT3 lipid remodeling activity, while noting that lysophosphatidylcholine species that serve as substrates for LPCAT3, also increase upon LXR activation^14^. Further studies will need to identify whether the axis LXR/LPCAT3 directly affects prostaglandin production in neurons. Thus, LXR may regulate lipid homeostasis in sensory neurons to, not only modulate ER stress-induced lipid overload, but modulate downstream pathways reliant on lipid intermediates and lipid balance between cytoplasm and membranes including prostaglandin synthesis.

Prostaglandins are also produced by non-neuronal cell types such as immune cells in the PNS in response to peripheral nerve injury and participating in nerve regenerative processes and pain^48,49^. In the past and in other models, LXR activation has been identified to block prostaglandin synthesis from immune cells, which may have interplaying mechanisms to attenuate pain via prostaglandin signaling among its various isoforms, especially PGE2 identified in Schwann cells and satellite glial cells upon nerve injury^50,51^. Interestingly, our findings did not identify changes in PGD2 levels in the spinal cord with i.p. injection of GW3965. The blood-spial cord barrier is highly restricted due to the absence of fenestrated capillary membranes and the low permeability of tight junctions^52^. Literature has shown that the rodent blood-spinal cord barrier is permeable to smaller proteins in circulation, such as albumin and inflammatory cytokines^53^. Without designated endothelial transport systems, it is possible that GW3965 is ineffective in passing this barrier via intraperitoneal injection. These complications warrant the use of intrathecal injections or nanoparticle delivery of GW3965 in future studies.

While lipidomics characterizes lipids from the whole neuron, the membrane, and most importantly, lipid rafts, largely make up the lipid content of neurons. LXR activation significantly decreased neuronal free fatty acids. Obese individuals are characterized as having high circulating levels of dietary free fatty acids, including palmitic acid, oleic acid, steric acid, and linoleic acid that contribute to the development of metabolic associated diseases and inflammation^9,54,55^. Given our observations, LXR activation may protect neurons from these insults. In maintenance of cholesterol homeostasis, GW3965 increases neuronal cholesterol ester content. Cholesterol esters are neutral substrates that are either sequestered into lipid droplets or lipoproteins for secretion^56^. LXR activation is known to promote cholesterol esterification to maintain cholesterol homeostasis, inducing target genes such as FASN and SREBP-1c that buffer intracellular cholesterol levels^57^. Future studies are required to dive further into the mechanisms behind LXR activation and lipid homeostasis in the PNS to downstream inflammatory pain pathways. Understanding these interplaying mechanisms may further our knowledge on the neurobiology behind obesity-induced neuropathic pain and provide effective therapeutic options for those who are affected.

## Methods

### Mice

All studies were conducted in accordance with recommendations in the Guide for the Care and Use of Laboratory Animals of the National Institutes of Health and the approval of the Loyola University Chicago Institutional Animal Care and Use Committee. All methods were carried out in accordance with relevant guidelines and regulations and reported in accordance with ARRIVE (Animal Research: Reporting of In Vivo Experiments) guidelines with experimenter blinded to the treatment. C57BL/6J (#000664) and RiboTag (#011029) mice were obtained from Jackson laboratory (Maine, USA) and crossed with transgenic mice carrying a Cre recombinase driven by the Scn10a promoter (Na_v_1.8::Cre mice) to generate wildtype and RiboTag+/+:Nav1.8Cre+/- mice. All mice were housed in a 12:12h light/dark cycle and were between 5 to 8 weeks of age at the beginning of experimental paradigms described below.

### Western diet

Wild type mice received either NC (Teklad LM-485) or WD (TD88137; contains 42%kcal from fat, 34% sucrose by weight, and 0.2% cholesterol) for 11 weeks or for 18 weeks beginning at 8 weeks of age (Envigo, Indiana, USA). Body weights were recorded weekly from the start of diet.

### Glucose and insulin tolerance tests

Mice were fasted overnight (12 hrs) and given an i.p. injection of glucose (1g/kg BW) after measurement of fasting glucose as performed previously^5^. After injection, blood glucose levels were measured using AlphaTrak glucometer (Fisher Scientific, Pennsylvania, USA) at different time points. For the insulin tolerance test, mice were fasted for 4 hrs and given an i.p injection of insulin (0.5U/kg BW) (Human-R Insulin U100, Lily). Blood glucose levels were measured before and after injection at different time points.

### Von Frey mechanical sensitivity

Mice were tested for mechanical allodynia under stimulation with von Frey filaments. Mice were acclimated in testing chambers for 1 hr before stimulation with 6 calibrated von Frey filaments (0.16;0.4;1;2;4;6;8 g) (North Coast Medical, California, USA). Filaments were applied for 1 s with 6 stimulations per filament with a 5 min break in between each filament as described previously^5^.

### Thermal nociception

Mice were tested for thermal hyperalgesia using Plantar Test Apparatus (Hargreaves Method) (IITC Life Science, California, USA) as described previously^5^. Mice were acclimated for 1 hr in testing chambers, and a cutoff time of 20 s was set for each thermal stimulation to avoid tissue damage if the animal had not responded.

### In vivo agonist treatment

Mice were treated with vehicle or LXR agonist (GW3965; 25mg/kg BW) as performed previously by i.p. injection twice a week for 7 weeks starting at 12 weeks of diet, where injections were 3 days apart^5^. Tissues were dissected and processed as detailed below.

### Transcript enrichment of Na_v_1.8

DRG from RiboTag+/+:Na_v_1.8Cre+/- mice were extracted to perform organotypic cultures followed by RNA isolation. Immunoprecipitation followed by mRNA purification was performed to isolate RNA associated with HA-tagged ribosomes of Na_v_1.8-expressing sensory neurons. Harvested DRG were homogenized in homogenization buffer and centrifuged at 0,000xg for 10 min at 4C. Supernatant was removed, and the homogenate was incubated at 4 °C with anti-HA antibody (Biolegend, #901513) at a 1:150 dilution for 4 hours on a tube rotator. Then, samples were transferred to a tube containing magnetic beads (Pierce A/G magnetic beads, California, USA) and incubated overnight at 4 °C on a tube spinner. The following day, supernatant from the samples were collected and the magnetic beads were washed with high salt buffer for 10 min at 4 °C on the spinner, repeated 3 times. After the final wash, lysis buffer (RNeasy Micro Kit, QIAGEN, Maryland, USA) and β-mercaptoethanol (10ul/mL) was added for mRNA elution. RNA was then isolated using RNeasy Micro Kit (QIAGEN, California, USA) following manufacturer’s instructions and quantified with Nanodrop (Thermo Fisher, Massachusetts, USA).

### Primary DRG neuronal culture

DRG from mice were harvested in ice-cold advanced DMEM and axotomized. Axotomized DRGs were then transferred and incubated in a collagenase A/trypsin mix (1.25mg/mL each) for 30 min. The digested DRG were then homogenized using fire polished glass pipettes and spun for 3 min at 3000xg. Supernatant was then removed, and cells were resuspended in DMEM/F12 containing 10% FBS. Cells were plated onto poly-l-lysine treated 12-well plates and maintained in a 37 °C and 5%CO2 incubator for 4 days with media changes supplemented with Ara-C (2 μM) to inhibit non-neuronal cell replication. Media was then replaced with DMEM/F12 containing2.5% FBS overnight before treatment. Following serum depletion, cells were treated with 250 μM palmitate for 24 hours, following with vehicle or 15 μM GW3965 in low serum media for another 24 hours. RNA was extracted using Acturus PicoPure RNA Extraction Kit (Applied Biosystems, California, USA).

### Quantitative PCR

100ng of RNA from DRG cultures was converted to cDNA using the High Capacity cDNA Reverse Transcription Kit (Applied Biosystems, California, USA). For all genes of interest, qPCR was performed using Sybr green-based assay using primers as shown in Table 1 (Roche, Indiana, USA) and IDT primers (IDT technologies, Iowa, USA).

**Table 1:** qPCR primer sequences.

Gene symbol	GeneBank No.		Primer sequence 5' → 3'
LDLR	NM_001252658.1	Forward	TCAGTCCCAGGCAGCGTAT
		Reverse	CTTGATCTTGGCGGGGTGTT
PTGDS	NM_008963.3	Forward	GGAGAAGAAAGCTGTATTGTATATGTGC
		Reverse	TAAAGGTGGTGAATTTCTCCTTCAG
ABCA1	NM_013454.3	Forward	CGTTTCCGGGAAGTGTCCTA
		Reverse	GCTAGAGATGACAAGGAGGATGGA
LPCAT3	NM_145130.2	Forward	TCTGGGGCAAATTTGTGCTG
		Reverse	AGCCACACTTTCATGTTGGC
IDOL	NM_153789.3	Forward	AGGAGATCAACTCCACCTTCT
		Reverse	ATCTGCAGACCGGACAGG
β-ACTIN	NM_007393.5	Forward	ACCTTCTACAATGAGCTGCG
		Reverse	CTGGATGGCTACGTACATGG

### Prostaglandin quantification

Harvested DRG were transferred to tubes prefilled with zirconium oxide beads with ice- cold PBS. The samples were homogenized using a Bullet Blender (Next Advance, New York, USA) at speed 8 for 5 min. Tissue homogenate was collected and centrifuged at 1000xg for 20 min at 4 °C. The supernatant was then collected and used for the ELISA assay. PGD2 levels in the DRG were evaluated using Prostaglandin D2-MOX ELISA Kit (Cayman Chemical, Michigan, USA) following the manufacturer’s instructions.

### Lipidomics

Primary DRG cultures were performed as described above. Cells were treated with vehicle or 15µM GW3965, with 5 biological replicates per treatment. Lipidomics was performed by UCLA Lipidomics Core using modified Bligh and Dyer lipid extraction for lipidomics analysis using The Sciex Lipidyzer Mass Spectrometry Platform. Samples were analyzed on the Sciex Lipidyzer Platform for quantitative measurement of 1100 lipid species across 13 lipid sub-classes. Data analysis was performed on Lipidyzer software. Quantitative values were normalized to the number of cells in the provided sample using Gene-Chip Mouse Gene 430.2 Arrays (Affymetrix, Santa Clara, CA).

### RNA sequencing

RNA was extracted from DRG of NC- or WD-fed mice or from primary DRG neurons cultured as described above and treated with vehicle or 15µM GW3965. Two biological replicates were used for each group. Total RNA was quantified by Qubit and quality assessed using Total RNA Pico Chip. RNA samples that passed this quality check were used as input for library construction. Samples were sequenced at the Northwestern Genomic Core (NuSeq).

### Statistical analysis

Statistical analysis for all experiments were done in GraphPad Prism 9. All data are represented as mean ± SEM. Comparisons between experimental treatments were performed using Two-sample t-test. *p*<0.05 was considered statistically significant.

## Supplementary Information

Supplementary Information 1.

Supplementary Information 2.

## Data Availability

The datasets generated during the current study are available in the Gene Expression Omnibus (GEO) repository. Accession numbers are as follows. NC vs. WD DRG RNA-seq data: GSE198895. Vehicle vs. GW3965 DRG primary RNA-seq data: GSE198898. Analyzed RNA- seq data are provided with this paper.

## References

[1] Callaghan B, Feldman E (2013). The metabolic syndrome and neuropathy: therapeutic 371challenges and opportunities. Ann. Neurol..

[2] Callaghan BC (2018). Diabetes and obesity are the main metabolic drivers of peripheral neuropathy. Ann. Clin. Transl. Neurol..

[3] Callaghan BC (2016). Metabolic syndrome components are associated with 375symptomatic polyneuropathy independent of glycemic status. Diabetes Care.

[4] Kazamel M, Stino AM, Smith AG (2021). Metabolic syndrome and peripheral neuropathy. Muscle Nerve.

[5] Gavini CK (2018). Liver X receptors protect dorsal root ganglia from obesity-induced endoplasmic reticulum stress and mechanical allodynia. Cell Rep..

[6] Hinder LM (2017). Dietary reversal of neuropathy in a murine model of prediabetes and the metabolic syndrome. Dis. Model. Mech..

[7] Jayaraj ND (2018). Reducing CXCR4-mediated nociceptor hyperexcitability reverses painful diabetic neuropathy. J. Clin. Invest..

[8] Shields SD (2012). Nav1.8 expression is not restricted to nociceptors in mouse peripheral nervous system. Pain.

[9] O'Brien PD (2019). Integrated lipidomic and transcriptomic analyses identify altered nerve triglycerides in mouse models of prediabetes and type 2 diabetes. Disease Models Mech..

[10] Stino AM, Smith AG (2017). Peripheral neuropathy in prediabetes and the metabolic syndrome. J. Diabetes Investig..

[11] McGregor BA (2018). Conserved transcriptional signatures in human and murine diabetic peripheral neuropathy. Sci. Rep..

[12] Sas KM (2018). Shared and distinct lipid-lipid interactions in plasma and affected tissues in a diabetic mouse model. J. Lipid Res..

[13] Laurencikiene J, Rydén M (2012). Liver X receptors and fat cell metabolism. Int. J. Obes..

[14] Rong X (2013). LXRs regulate ER stress and inflammation through dynamic modulation of membrane phospholipid composition. Cell Metab..

[15] Rong X (2015). Lpcat3-dependent production of arachidonoyl phospholipids is a key determinant of triglyceride secretion. eLife.

[16] Hashidate-Yoshida T (2015). Fatty acid remodeling by LPCAT3 enriches arachidonate in phospholipid membranes and regulates triglyceride transport. eLife.

[17] Ricciotti E, FitzGerald GA (2011). Prostaglandins and inflammation. Arterioscler. Thromb. Vasc. Biol..

[18] Jang Y, Kim M, Hwang SW (2020). Molecular mechanisms underlying the actions of arachidonic acid-derived prostaglandins on peripheral nociception. J. Neuroinflamm..

[19] Breyer RM, Bagdassarian CK, Myers SA, Breyer MD (2001). Prostanoid receptors: subtypes and signaling. Annu. Rev. Pharmacol. Toxicol..

[20] Tavares-Ferreira D (2020). Sex differences in nociceptor translatomes contribute to divergent prostaglandin signaling in male and female mice. Biol. Psychiatry.

[21] Jenkins DW, Feniuk W, Humphrey PP (2001). Characterization of the prostanoid receptor types involved in mediating calcitonin gene-related peptide release from cultured rat trigeminal neurones. Br. J. Pharmacol..

[22] Li N (2010). LXR modulation blocks prostaglandin E2 production and matrix degradation in cartilage and alleviates pain in a rat osteoarthritis model. Proc. Natl. Acad. Sci. U.S.A.

[23] Thomas DG (2018). LXR Suppresses inflammatory gene expression and neutrophil migration through cis-repression and cholesterol efflux. Cell Rep..

[24] Bonomo RR (2020). Fecal transplantation and butyrate improve neuropathic pain, modify immune cell profile, and gene expression in the PNS of obese mice. Proc. Natl. Acad. Sci..

[25] Chen YI, Wei PC, Hsu JL, Su FY, Lee WH (2016). NPGPx (GPx7): a novel oxidative stress sensor/transmitter with multiple roles in redox homeostasis. Am. J. Transl. Res..

[26] Cha HN, Woo CH, Kim HY, Park SY (2021). Methionine sulfoxide reductase B3 deficiency inhibits the development of diet-induced insulin resistance in mice. Redox. Biol..

[27] Bosma M (2014). Sequestration of fatty acids in triglycerides prevents endoplasmic reticulum stress in an in vitro model of cardiomyocyte lipotoxicity. Biochim. et Biophys. Acta BBA Mol. Cell Biol. Lipids.

[28] Zhou Y (2012). OSBP-related proteins (ORPs) in human adipose depots and cultured adipocytes: evidence for impacts on the adipocyte phenotype. PLoS ONE.

[29] Asai M (2013). Loss of function of the melanocortin 2 receptor accessory protein 2 is associated with mammalian obesity. Science.

[30] Chaly AL, Srisai D, Gardner EE, Sebag JA (2016). The Melanocortin receptor accessory protein 2 promotes food intake through inhibition of the prokineticin receptor-1. eLife.

[31] Johnson AR (2016). Metabolic reprogramming through fatty acid transport protein 1 (FATP1) regulates macrophage inflammatory potential and adipose inflammation. Mol. Metab..

[32] Flowers MT (2008). Liver gene expression analysis reveals endoplasmic reticulum stress and metabolic dysfunction in SCD1-deficient mice fed a very low-fat diet. Physiol. Genom..

[33] Zelcer N, Hong C, Boyadjian R, Tontonoz P (2009). LXR regulates cholesterol uptake through Idol-dependent ubiquitination of the LDL receptor. Science.

[34] Pfeuffer M, Jaudszus A (2016). Pentadecanoic and Heptadecanoic acids: Multifaceted odd-chain fatty acids. Adv. Nutr. Int. Rev. J..

[35] Kazachkov M, Chen Q, Wang L, Zou J (2008). Substrate preferences of a lysophosphatidylcholine acyltransferase highlight its role in phospholipid remodeling. Lipids.

[36] Law S-H (2019). An updated review of lysophosphatidylcholine metabolism in human diseases. Int. J. Mol. Sci..

[37] Gregg EW (2004). Prevalence of lower-extremity disease in the US adult population >=40 years of age with and without diabetes: 1999–2000 national health and nutrition examination survey. Diabetes Care.

[38] Tesfaye S (2011). Painful diabetic peripheral neuropathy: Consensus recommendations on diagnosis, assessment and management. Diabetes Metab. Res. Rev..

[39] Stino AM, Rumora AE, Kim B, Feldman EL (2020). Evolving concepts on the role of dyslipidemia, bioenergetics, and inflammation in the pathogenesis and treatment of peripheral neuropathy. J. Peripher. Nerv. Syst..

[40] Rumora AE (2018). Dyslipidemia impairs mitochondrial trafficking and function in sensory neurons. FASEB J..

[41] Hinder LM, Vivekanandan-Giri A, McLean LL, Pennathur S, Feldman EL (2013). Decreased glycolytic and tricarboxylic acid cycle intermediates coincide with peripheral nervous system oxidative stress in a murine model of type 2 diabetes. J. Endocrinol..

[42] Listenberger LL (2003). Triglyceride accumulation protects against fatty acid-induced lipotoxicity. Proc. Natl. Acad. Sci..

[43] Plataki M (2019). Fatty acid synthase downregulation contributes to acute lung injury in murine diet-induced obesity. JCI Insight.

[44] Ellis A, Bennett DL (2013). Neuroinflammation and the generation of neuropathic pain. Br. J. Anaesth..

[45] Kampoli AM (2011). Potential pathogenic inflammatory mechanisms of endothelial dysfunction induced by type 2 diabetes mellitus. Curr. Pharm. Des..

[46] Ebersberger A (2011). Effects of prostaglandin D2 on tetrodotoxin-resistant Na+ currents in DRG neurons of adult rat. Pain.

[47] Boyd JT (2021). Elevated dietary ω-6 polyunsaturated fatty acids induce reversible peripheral nerve dysfunction that exacerbates comorbid pain conditions. Nat. Metab..

[48] Forese MG (2020). Prostaglandin D2 synthase modulates macrophage activity and accumulation in injured peripheral nerves. Glia.

[49] Trimarco A (2014). Prostaglandin D2 synthase/GPR44: A signaling axis in PNS myelination. Nat. Neurosci..

[50] Muja N, Devries GH (2004). Prostaglandin E2 and 6-keto-prostaglandin F1? production iselevated following traumatic injury to sciatic nerve. Glia.

[51] Capuano A (2009). Proinflammatory-activated trigeminal satellite cells promote neuronal sensitization: Relevance for migraine pathology. Mol. Pain.

[52] Bartanusz V, Jezova D, Alajajian B, Digicaylioglu M (2011). The blood-spinal cord barrier: Morphology and clinical implications. Ann. Neurol..

[53] Winkler EA, Sengillo JD, Bell RD, Wang J, Zlokovic BV (2012). Blood-spinal cord barrier pericyte reductions contribute to increased capillary permeability. J. Cereb. Blood Flow Metab..

[54] Feng R (2017). Free fatty acids profile among lean, overweight and obese non-alcoholic fatty liver disease patients: a case – control study. Lipids Health Dis..

[55] Feldman EL, Nave KA, Jensen TS, Bennett DLH (2017). New horizons in diabetic neuropathy: Mechanisms, bioenergetics, and pain. Neuron.

[56] Luo J, Yang H, Song BL (2020). Mechanisms and regulation of cholesterol homeostasis. Nat. Rev. Mol. Cell Biol..

[57] Zhu R, Ou Z, Ruan X, Gong J (2012). Role of liver X receptors in cholesterol efflux and inflammatory signaling (review). Mol. Med. Rep..

